# Primary Leiomyoma of the Liver: A Review of a Rare Tumour

**DOI:** 10.1155/2014/959202

**Published:** 2014-11-19

**Authors:** Ayodeji Oluwarotimi Omiyale

**Affiliations:** Department of General Surgery, Royal Shrewsbury Hospital, Shrewsbury SY3 8XQ, UK

## Abstract

*Context.* Primary leiomyoma of the liver is a rare tumour with uncertain pathogenesis with similar presentation with other tumours of the liver. Little is known about its clinical course. *Objectives.* To review the literature for case reports of primary leiomyoma of the liver. *Methods.* Extensive literature search was carried out for case reports of primary leiomyoma of the liver. *Results.* A total of 36 cases of primary leiomyoma of the liver were reviewed. The mean age of presentation is 43 years with slight female sex affectation; females accounted for 55.6% of the cases reported in the literature. The average size of the tumour is 8.7 cm. 34.4% of the cases reviewed were incidental finding with the mean follow-up time of 33 months with most cases reporting no evidence of disease. *Conclusions.* Primary leiomyoma of the liver is very rare tumour with complex pathogenesis which remains largely unknown. Imaging of the tumour does not allow for a tissue specific diagnosis; hence histological review of the tissue specimen and immunohistochemical stains are imperative for diagnosis. Surgical resection is both diagnostic and curative. The diagnosis of primary leiomyoma of the liver should be considered as a differential in the management of liver tumours.

## 1. Introduction

Leiomyoma is a benign smooth muscle neoplasm of mesenchymal origin which commonly occurs in the genitourinary system and the gastrointestinal tract of the body but which rarely occurs in the liver [1, 2]. The first case report of primary leiomyoma of the liver was first described in a 42-year-old woman by Demel in 1926 [3].

This paper seeks to review primary leiomyoma of the liver in the literature because of its rarity, unclear pathogenesis, and the diagnostic challenges it poses in clinical practice.

## 2. Methods

Case reports and case series of primary leiomyoma of the liver were retrieved by extensive literature search of PubMed, Ovid SP, Cochrane database of systematic reviews, Embase, and Clinical Evidence Online. Further search of the literature was carried out by manually searching the relevant references of the studies retrieved. The inclusive criteria include relevant publications of primary leiomyoma of the liver and hence studies with coexisting leiomyoma in other parts of the body were excluded.

Epidemiologic, pathologic, clinical, imaging, and prognostic data were retrieved and assessed for all studies. The search keywords include primary hepatic leiomyoma, primary leiomyoma of the liver, primary benign lesions of the liver, and primary tumours of the liver.

## 3. Results

The clinical and pathologic characteristics of the 35 cases reviewed with the treatment and the clinical outcome are outlined in Table 1.

## 4. Discussion

Primary leiomyomas of the liver are very rare tumours. Eighty-seven years after the first case of primary leiomyoma of the liver was reported, to the best knowledge of the author, 36 cases of primary leiomyoma of the liver have so far been reported in the literature.

Hawkins et al. [4] in 1980 proposed criteria that must be met for the diagnosis of primary liver leiomyoma. The tumour must be composed of leiomyocytes. Secondly, the presence of leiomyoma in other sites of the body like uterus and the gastrointestinal tract must be excluded. If the uterus is surgically absent, the diagnosis of primary leiomyoma of the liver must not be made without the review of the report and sections from the hysterectomy.

### 4.1. Epidemiology

Primary leiomyomas of the liver have been reported in both paediatric and adult populations. There are reports in the literature to suggest the incidence of the tumour in both immunocompetent and immunosuppressed patients. The mean age of presentation is 43 years (range 4.6–87). Primary leiomyomas of the liver have been reported to have female sex predilection [2, 5–7]. Luo et al. suggested that the observed female preponderance may be partly due to the activity of the smooth muscle cells in female urogenital tissue in carcinogenesis [2]. However this view seems to contradict one of the main diagnostic criteria for primary leiomyoma of the liver as proposed by Hawkins et al. [4] which seeks to exclude leiomyoma in other parts of the body especially in the urogenital tissue.

This review of 36 cases however demonstrates slight female sex affectation with females accounting for 55.6% of the cases. Familial predispositions have not been reported. The distribution of the lesion is equal in both right and left lobes of the liver with two cases involving the caudate lobe of the liver [8, 9].

### 4.2. Pathogenesis

The pathogenesis of primary leiomyoma of the liver is not clear and largely unknown. Some theories have emerged as to the possible pathogenesis of these tumours. Proliferations of smooth muscle of the hepatic vessels or the biliary tree have been suggested as a possible origin [2, 10–13]. However the argument against origin from the bile ducts is that large extra hepatic ducts have very few smooth cells [11, 14].

Immunosuppressive states which include either posttransplant patients on immunosuppressive therapy or patients with human immunodeficiency virus (HIV) have been suggested as a possible causal factor in primary leiomyoma of the liver. Increased risks of de novo neoplasia after transplantation are well documented in the literature [15–17].

Possible explanations for the susceptibility of immunocompromised patients to neoplasms include the disruption of the immunosurveillance ability of the host with the subsequent development of the tumours that would otherwise have been suppressed by a normal immune system [18]. The incidence of primary leiomyoma of the liver within the setting of immunosuppression in this review which accounts for 39.3% of the cases appears to be in support of this theory [2, 10, 18–25]. Immunosuppression alone does not totally explain the pathogenesis of this tumour because of the incidence of the tumour in immunocompetent individuals.

The theory of the possible role of viral induced oncogenesis has been suggested. This is because of the evidence that implicates some DNA viruses in the aetiology of some neoplasms particularly Epstein-Barr virus in smooth muscle tumours [16, 18]. This theory is further supported by the observation that patients with immunosuppression are at high risks of developing virus-associated neoplasms although the exact mechanism is not known [18, 26].

A possible explanation is a multistep theory of viral oncogenesis which suggests that virus infected cells undergo an uncontrolled polyclonal proliferation in the setting of the reduced immune surveillance of the viral transformed cells. Further cytogenetic events alter the growth regulation of a subset of cells, leading to a monoclonal expansion of tumour cells [18]. In support of this theory is the observation that 5 cases were reported to be positive for EBV in this review and the 5 cases were also in the setting of immunosuppression [10, 18–20, 22]. The fact that other case reports in this review were not associated with Epstein-Barr virus suggests a rather complex pathogenesis for primary leiomyoma of the liver. Epstein-Barr viral oncogenesis alone does not explain the pathogenesis. Virus associated tumours have been observed to exhibit different range of differentiation from well differentiated to poorly differentiated and some may show features suggestive of leiomyosarcomas [27]. The risk of other cancers in patients with immunosuppression not linked to viruses is also increased [28–30].

### 4.3. Clinical Features

The clinical presentation of primary leiomyoma of the liver is similar to the presentation of other liver neoplasms. The most common clinical symptom in this review is abdominal, epigastric, or right upper quadrant pain which accounts for 42.4% of cases reported [1–3, 5, 11, 13, 19–21, 31–33]. 33.3% of the cases were incidental with one of them an incidental finding at autopsy [10]. Other clinical features include abdominal mass [4, 7, 12, 34, 35], abdominal discomfort [22], dyspepsia [36], and liver dysfunction [6].

Primary leiomyoma of the liver may rarely present as a composite tumour. Yanase et al. reported the case of a 59-year-old lady with 13 × 10 × 9 cm firm tumour with mainly a solid tissue portion and interconnected multilocular cystic lesions. Histologic diagnosis of primary leiomyoma of the liver encasing hepatobiliary cystadenoma was made [6].

**Figure 1:**
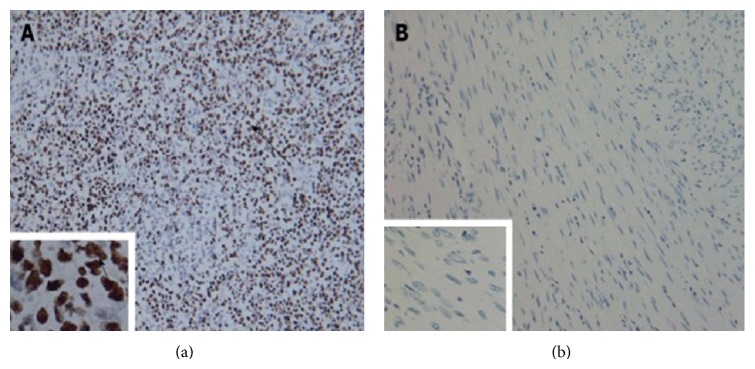
Tumour cells stained positive by* in situ* hybridization with Epstein-Barr virus-encoded small RNA. (a) Positive control staining ×200, ×1000; (b) tumour cell staining ×200, ×1000. Arrows indicate positive staining of the nuclei. Reprinted from [2] with the permission of the Director, Editorial Office of the World Journal of Gastroenterology.

Screening for tumour markers alpha fetoprotein, carbohydrate antigen 19-9 and carcinoembryonic antigen are usually negative [11, 12, 19]. Serological testing for EBV combined with in situ hybridization indicates the tumour cells positive for EBV encoded small RNA (Figure 1) [2, 10, 18, 20]. In situ hybridization is the gold standard for the detection and localization of latent EBV in tissues [2].

### 4.4. Imaging

Imaging alone does not show tissue specific diagnosis and cannot reliably differentiate between primary leiomyoma and other differential diagnosis like leiomyosarcoma, hepatocellular adenoma, hepatocellular carcinoma, angiomyolipoma, and hypervascular metastatic lesions [36, 37]. There is no notable difference in the imaging of patients with or without immunosuppression [19].

### 4.5. CT

**Figure 2:**
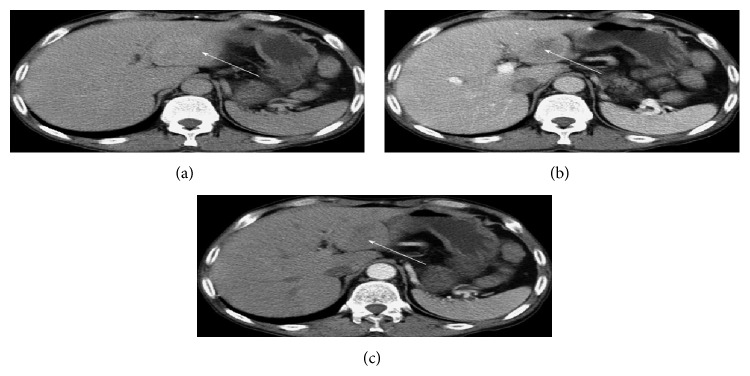
CT Abdomen with a mass in segment III of the liver in the hepatic equilibrium (a), portal venous (b), and hepatic arterial phase (c). Reprinted from [2] with the permission of the Director, Editorial Office of the World Journal of Gastroenterology.

CT findings in leiomyoma of the liver have been variously reported in the cases reviewed as hypodense lesions with strong enhancement in both arterial and portal phase [5, 11, 13, 21, 23, 36, 38] with some reports describing peripheral rim enhancement [8, 37]. An increased enhancement in the arterial phase and a sustained homogeneous enhancement in both hepatic venous and equilibrium phases have also been reported [39] (Figure 2).

### 4.6. Ultrasound

Ultrasound findings in primary leiomyoma of the liver in the literature have been described as hypoechoic lesions with varying degrees of heterogeneity [5, 8, 11, 36, 37]. Perini et al. reported heterogeneous mass displacing the inferior vena cava (IVC) and the right kidney medially across the midline [19].

### 4.7. MRI

MRI findings from several studies suggest hypointense lesions on T1-weighted MRI images with hyperintense lesions on T2-weighted sequences with inhomogeneous contrast uptake [8, 19, 20, 37]. However hypointense lesions in the T2-weighted MRI images have also been reported which the authors associated with the dense fusocellular nature of the tumour [36].

The development of liver specific MR contrast agents which includes reticuloendothelial system specific contrast agents and hepatocytes specific contrast agents have been shown to potentially improve the detection and characterization of liver lesions by providing functional and morphologic information of the liver simultaneously [39–41]. Gadobenate dimeglumine is a gadolinium-based contrast agent that is partially taken up by functioning liver cells and excreted without biotransformation through the biliary duct system. Gadobenate dimeglumine shows a vascular-interstitial distribution in the first minutes after bolus injection. Normal liver and benign liver lesions show increased signal intensity on T1-weighted MR images during the delayed liver-specific phase because of active contrast uptake by functioning hepatocytes. The absence of contrast retention during the liver-specific phase is believed to be indicative of malignant liver lesions [39].

It has also been suggested that liver specific MR contrast agents may be misleading in the diagnosis of primary leiomyoma of the liver. The absence of contrast retention during the liver specific contrast enhanced MRI, in the case report, led them to suspect a malignant lesion of the liver but it turned out to be a primary leiomyoma on histology after surgical resection [39]. This finding is consistent with earlier reports in the literature that demonstrated equivocal appearance of primary leiomyoma of the liver after the administration of liver specific contrast agents [36, 41].

### 4.8. Angiography

Angiography has been reported to demonstrate irregular [37], marginal [8], or diffuse [5] hypervascularity. Hawkins also reported a selective angiogram through the left hepatic artery which demonstrated abnormal mass effect, stretching of the feeding vessels, and scattered pooling throughout the tumour. The authors concluded that the angiography study was nondiagnostic [4].

### 4.9. Preoperative Diagnosis

Attempts have been made to make a preoperative diagnosis of this tumour so as to prevent unwarranted diagnostic surgical procedures. CT guided fine needle biopsy had reportedly failed to determine the nature of the mass despite the fact that the primary leiomyoma of the liver in this case was the largest ever reported in the literature with a size of 30 cm. The patient underwent extended right hepatectomy. No extra-hepatic lesions were seen at surgery and the uterus was normal [12]. Percutaneous biopsy was attempted with mixed outcomes in the two cases reported by Sadler et al. [21]. One reported “well differentiated smooth muscle neoplasm consistent with hepatic leiomyoma” while multiple attempts at percutaneous biopsy were not successful in the second case [21].

Sousa et al. initially performed a US-guided fine needle aspiration (FNA) which was inconclusive because of because of insufficient sample which included only a small group of normal looking hepatocytes [36]. The histological review of the 18G Trucut biopsy sample taken by Sousa et al. proved to be accurate in the diagnosis of leiomyoma of the liver which was further confirmed after surgical resection [36]. This accurate preoperative diagnosis from biopsy sample is consistent with other reports in the literature [24, 25].

The inconclusive FNA report from the case reported by Sousa et al. is consistent with the series reported by Guy et al. who further reiterated the difficulty of getting adequate sample in 10% of cases where FNA was used in the diagnosis of spindle cell lesions of the liver [42]. Hence FNA does not seem appropriate and adequate for the diagnosis of primary leiomyoma of the liver.

### 4.10. Macroscopic Features

**Figure 3:**
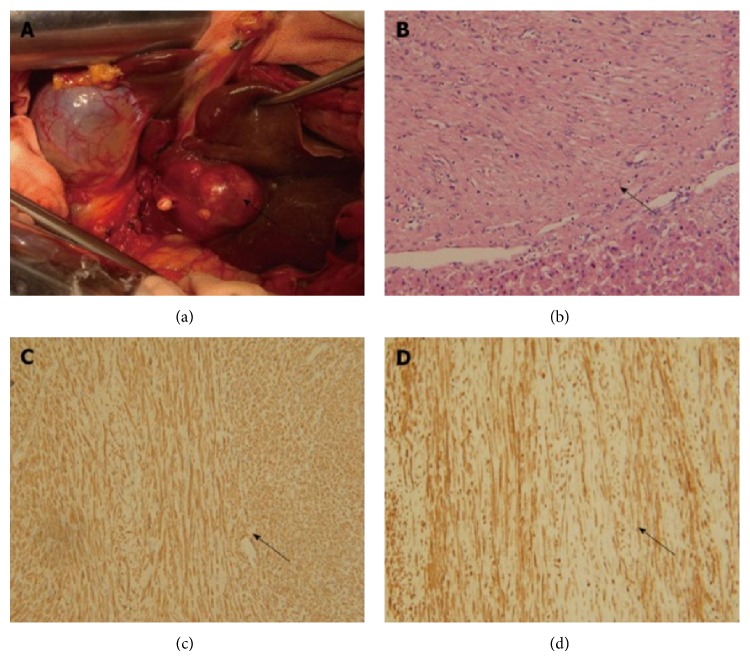
Pathologic features. (a) Intraoperative feature of primary leiomyoma of the liver. (b) Tumour (arrow) and normal liver tissue, H&E staining, ×200; (c) *α*-smooth muscle actin staining (arrow) of tumour tissues, immunohistochemical staining, ×200; (d) Desmin staining (arrow) of tumour tissues, immunohistochemical staining, ×200. Reprinted from [2] with the permission of the Director, Editorial Office of the World Journal of Gastroenterology.

The average size of the tumour in this review is 8.7 cm (range 2–30). The largest size of this tumour in the literature, 30 cm, was reported by Belli et al. in a 67-year-old woman who presented with abdominal mass [12]. Primary Leiomyoma of the liver has been described in the literature as a solitary firm, white, fasciculate, and well demarcated tumour which is consistent with the findings of this review [4, 10, 19, 39]. One author reported a case of primary leiomyoma of the liver with two sharply delineated tumours [20] but other cases in the review have been reported as solitary tumours. The shape has been reported to be roughly spherical [20] to oval [19] (Figure 3(a)).

### 4.11. Microscopic Features

Histological review of tissue sections and specimens is absolutely important because the distinction between benign and malignant smooth muscle tumours of the gastrointestinal tract on imaging is not very clear [12].

The cellular architecture has been variably described as multiple interlacing bundles of uniform spindle cells [39] homogeneous pattern of interlacing bundles of uniform elongate cells with a plump spindle shaped [4], whirling bundles of well differentiated regular spindle shaped smooth muscle cells [2, 12], and highly cellular population of spindle cells arranged in interwoven fascicles (Figure 3(b)). Cells have slightly eosinophilic [20] to abundant eosinophilic cytoplasm [10, 24]. High density reticular fibres with a peripheral collagen rich zone which indicates expanding growth have been reported [20, 24]. Electron microscopy findings suggest tumour cells with well-defined basement membrane, scattered electron dense condensations in the plasma membrane, abundant glycogen, and pinocytotic vesicles and cytoplasmic filaments [24].

Central [19, 25] and focal [24] areas of necrosis have been described in the case reports. However it was not stated in the case reports if the necrosis were coagulative in nature.

Primary leiomyomas of the liver have been largely reported without evidence of mitotic changes except for few case reports which variously reported scarce, low, and rare mitoses [2, 4, 6, 12, 18, 20, 21, 24]. Histological features suggestive of malignancy include prominent cellular atypia with nuclear pleomorphism, large size, presence of infiltration, dense cellularity, degenerative changes, areas of coagulative necrosis, and increased mitotic rates (more than 1/10 HPF) [1, 12, 18, 19].

Mitotic index as defined by the number of mitoses per specified high power field was not documented for some of the case reports [2, 12, 21]. Doyle et al. [24] and Hawkins et al. [4] reported mitotic count of less than 1/10 HPF (high power field). Davidoff et al. [18] and Sclabas et al. [20] reported a mitotic count of 1/50 HPF and less than 1/20 HPF, respectively. Various mitotic indexes have been suggested as a cut off criteria for leiomyosarcoma in nonuterine smooth muscle tumours. Ranchod and Kempson [43] suggested five or more mitoses/10 HPF while between 5–10/50 HPF has been suggested by some authors [44–46]. The use of mitotic index in a uniform manner for all cases reported in the future is needful to ensure unequivocal diagnosis especially in a case reported with mild cellular atypia in the presence of mitoses [21] which may suggest a distinct possibility of malignancy.

### 4.12. Immunohistochemistry

The cells in the case reports reviewed have been reported to be positive to alpha smooth muscle actin (Figure 3(c)). Some of the cells also stained positive to Desmin (Figure 3(d)) [2, 24] and Vimentin [12]. There is lack of expression of CD 34 [20, 39], CD 68, Vimentin [10] HMB-45, S100 [19, 20], CD 117, and DOG1 [2]. Ultrastructural studies have been reported to show filaments, some dense bodies, and a few pinocytic vesicles [4]. The immunohistochemical stains are useful in ruling out possible differential diagnosis. CD 117, CD34, and DOG1 are markers of gastrointestinal stromal tumours (GIST) and HMB-45 reactivity suggests angiomyolipoma [5, 36, 47].

### 4.13. Treatment

Primary leiomyoma of the liver is amenable to surgery. Surgical resection of the tumour appears to be both diagnostic and curative in this review of the literature. The prognosis of this tumour appears to be excellent without evidence of disease during the follow-up of the cases. The average follow-up of the cases in this review is 33 months (range 4–108).

## 5. Conclusion

This paper to the best of the knowledge of the author is the largest review of case reports of primary leiomyoma of the liver in the literature. Primary leiomyoma of the liver is a very rare tumour with a complex pathogenesis which remains largely unknown. The diagnosis of the primary leiomyoma of the liver must meet a set of diagnostic criteria proposed by Hawkins et al. which ensures the cells are leiomyocytes and the exclusion of coexisting leiomyoma from other sites of the body. Metastatic workup to exclude occult leiomyoma elsewhere should be undertaken. This should include investigations like oesophagogastroduodenoscopy, colonoscopy, imaging techniques like CT scans and MRI, and a thorough exploration during surgery.

Primary leiomyoma of the liver should be considered as a differential diagnosis of liver lesions with or without immunosuppression. Multiple imaging techniques do not allow for a tissue specific diagnosis; hence histological review of the tissue specimen and immunohistochemical stains are imperative for diagnosis. Surgical resection is both diagnostic and curative.

## Figures and Tables

**Table 1:** Clinical and pathologic features of the reviewed cases of primary leiomyoma of the liver.

Cases	Age	Sex	Clinical features	Size (cm)	Location	EBV status	Mitosis	Immunosuppression	Necrosis	Tx	F/U (Mths)	Outcome
Perini et al. [19]	45	M	Epigastric pain	4.3	LL	Positive	Nr	Yes	nr	sectionectomy	4	ned
Davidoff et al. [18]	5	M	Incidental	15	RL	Positive	Low	Yes	nr	R trisegmentectomy	10	ned
Cheuk et al. [22]	37	M	Abdominal discomfort	3.5	LL	Positive	Nr	Yes	nr	conservative	nr	nr
Prevot et al. [10]	33	M	Incidental (autopsy)	2	RL	Positive	None	Yes	None	no surgical tx	0	D
Sclabas et al. [20]	30	F	Epigastric pain	4.4, 0.6	LL	Positive	Low	Yes	nr	LL hepatectomy	30	ned
Luo et al. [2]	48	M	RUQ pain	4.9	LL	Negative	Low	Yes	nr	LL hepatectomy	24	ned
Raber et al. [23]	46	F	Incidental	2.8	RL	Nr	None	Yes	nr	conservative	84	ned
Doyle et al. [24]	5	F	Incidental	3	LL	Nr	Low	Yes	Yes	LL segmentectomy	8	ned
Ha et al. [25]	9	M	Incidental	5.6	LL	Nr	Nr	Yes	Yes	LL hepatectomy	nr	ned
Yoon et al. [34]	41	F	RUQ mass	19	RL	Nr	Nr	None	nr	RL hepatectomy	nr	nr
Yanase et al. [6]	59	F	Liver dysfunction	13	RL	Nr	Low	None	None	RL hepatectomy	21	ned
Beuzen et al. [5]	36	F	RUQ pain	5	LL	Nr	None	None	None	Bi segmentectomy	108	ned
Santos et al. [13]	28	F	RUQ pain	5.5	RL	None	None	None	None	segmentectomy	36	ned
Urizono et al. [8]	71	M	Incidental	3	RL	Nr	None	None	nr	caudate lobectomy	nr	ned
Perini et al. [19]	45	F	RUQ pain	20	RL	Nr	None	None	Yes	segmentectomy	72	ned
Marin et al. [39]	64	F	Incidental	3	RL	Nr	None	None	nr	R hepatectomy	12	ned
Belli et al. [12]	67	F	Abdominal mass	30	RL	Nr	Low	None	nr	ER hepatectomy	48	ned
Sousa et al. [36]	61	F	Dyspepsia	9.5	LL	Nr	None	None	nr	LL hepatectomy	16	ned
Hollands et al. [11]	17	M	Epigastric pain	9	LL	Nr	None	None	nr	LL hepatectomy	12	ned
Kanazawa et al. [37]	31	M	Incidental	3.5	LL	Nr	None	None	nr	LL segmentectomy	nr	nr
Reinertson et al. [1]	32	F	RUQ pain	10	LL	Nr	None	None	nr	LL hepatectomy	24	ned
kalil et al. [7]	44	F	RUQ mass	7	RL	Nr	Nr	None	nr	atypical resection	nr	nr
Imasato et al. [9]	61	F	Incidental	4.5	RL	Nr	Nr	None	nr	RL hepatectomy	nr	nr
Hawkins et al. [4]	66	M	Abdominal mass	13	LL	Nr	Low	Nr	nr	LL hepatectomy	48	ned
Herzberg et al. [31]	30	F	RUQ pain	19	RL	Nr	Nr	Nr	nr	R hepatectomy	nr	nr
Ishak and Rabin [35]	64	M	Abdominal mass	Nr	RL	Nr	Nr	Nr	nr	laparotomy	nr	nr
Demel [3]	42	F	RUQ pain	12	RL	Nr	Nr	Nr	nr	laparotomy	nr	nr
Mesenas et al. [48]	59	M	Incidental	3.6	RL	Nr	Nr	Nr	nr	segmentectomy	nr	nr
Rummeny et al. [33]	46	F	RUQ pain	Nr	Nr	Nr	Nr	Nr	nr	nr	nr	nr
Tan et al. [38]	31	F	Nr	Nr	Nr	Nr	None	None	nr	hepatic resection	nr	nr
Tan et al. [38]	42	M	Nr	Nr	Nr	Nr	None	None	nr	hepatic resection	nr	nr
Tan et al. [38]	69	M	Nr	Nr	Nr	Nr	None	None	nr	hepatic resection	nr	nr
Sadler et al. [21]	36	M	Abdominal pain	Nr	LL	Nr	Low	Yes	None	hepatic resection	nr	nr
Sadler et al. [21]	36	M	Abdominal pain	Nr	LL	Nr	Nr	Yes	nr	conservative	nr	nr
Bartoli et al. [49]	34	F	Incidental	Nr	RL	Nr	Nr	Nr	nr	RL hepatectomy	nr	nr
Rios-Dalenz [32]	87	F	RUQ pain	Nr	LL	Nr	Nr	Nr	nr	no surgical tx	nr	nr

Nr: not reported; ned: no evidence of disease.
